# Multiple Cross Displacement Amplification Combined With Real-Time Polymerase Chain Reaction Platform: A Rapid, Sensitive Method to Detect *Mycobacterium tuberculosis*

**DOI:** 10.3389/fmicb.2021.812690

**Published:** 2021-12-23

**Authors:** Wei-wei Jiao, Gui-rong Wang, Lin Sun, Jing Xiao, Jie-qiong Li, Ya-cui Wang, Shu-ting Quan, Hai-rong Huang, A-dong Shen

**Affiliations:** ^1^Key Laboratory of Major Diseases in Children, Beijing Key Laboratory of Pediatric Respiratory Infection Disease, Ministry of Education, National Key Discipline of Pediatrics (Capital Medical University), National Clinical Research Center for Respiratory Diseases, National Center for Children’s Health, Beijing Pediatric Research Institute, Beijing Children’s Hospital, Capital Medical University, Beijing, China; ^2^National Tuberculosis Clinical Laboratory, Beijing Key Laboratory for Drug Resistance Tuberculosis Research, Beijing Tuberculosis and Thoracic Tumor Research Institute, Beijing Chest Hospital, Capital Medical University, Beijing, China; ^3^Children’s Hospital Affiliated to Zhengzhou University, Henan Children’s Hospital, Zhengzhou Children’s Hospital, Zhengzhou, China

**Keywords:** *Mycobacterium tuberculosis*, multiple cross displacement amplification, MCDA, diagnosis, pulmonary tuberclosis

## Abstract

In this study, we evaluated the diagnostic accuracy of multiple cross displacement amplification (MCDA) combined with real-time PCR platform in pulmonary tuberculosis (PTB) patients. Total 228 PTB patients and 141 non-TB cases were enrolled. Based on the analysis of the first available sample of all participants, MCDA assay showed a higher overall sensitivity (64.0%), with a difference of more than 10% compared with Xpert MTB/RIF (Xpert) assay (51.8%, *P* < 0.05) and combined liquid and solid culture (47.8%, *P* < 0.001) for PTB diagnosis. In particular, MCDA assay detected 31 probable TB patients, which notably increased the percentage of confirmed TB from 57.9% (132/228) to 71.5% (163/228). The specificities of microscopy, culture, Xpert and MCDA assay were 100% (141/141), 100% (141/141), 100% (141/141), and 98.6% (139/141), respectively. Among the patients with multiple samples, per patient sensitivity of MCDA assay was 60.5% (52/86) when only the first available sputum sample was taken into account, and the sensitivity increased to 75.6% (65/86) when all samples tested by MCDA assay were included into the analysis. Therefore, MCDA assay established in this study is rapid, accurate and affordable, which has the potential in assisting the accurate and rapid diagnosis of PTB and speed up initiation of TB treatment in settings equipped with real-time PCR platform.

## Introduction

Tuberculosis (TB), caused by *Mycobacterium tuberculosis* (MTB), is still a leading cause of mortality worldwide from a single infectious agent, being responsible for 9.9 million new cases and 1.3 million deaths in 2019 (World Health Organization [WHO], 2021). China is one of the high TB burden countries, accounting for 8.5% of all estimated incident cases globally (World Health Organization [WHO], 2021). Thus, much effort should be made to achieve the global END TB targets.

Rapid and accurate diagnosis is essential for TB control, which facilitates the timely initiation of appropriate treatment and further decreases the transmission risk. Current diagnostic tests for TB disease include smear microscopy, culture and rapid molecular tests. Smear microscopy is a routinely available test with low sensitivity. Culture-based methods remain the reference standard. However, it takes up to 8 weeks to provide the results and cannot meet the clinical needs. The Xpert MTB/RIF (Xpert) assay and loop-mediated isothermal amplification test (TB-LAMP) is World Health Organization (WHO) endorsed molecular tests (World Health Organization [WHO], 2011, 2016, 2017). Xpert is an automated real-time polymerase chain reaction (PCR) test with sensitivity comparable to liquid culture (Boehme et al., 2010) and is already widely used. The next-generation test Xpert Ultra has higher sensitivity for MTB detection, but it is not a routine laboratory test in China until now. Both Xpert and Xpert Ultra rely on GeneXpert machines and expensive consumable cartridges, which cannot be affordable in resource-poor settings. TB-LAMP is a promising technique with relatively simple device demand. Even a water bath could meet the needs of nucleic acid amplification. However, the results of TB-LAMP were usually determined by unaided eyes or under ultraviolet light, which is potentially subjective and make ambiguous judgment (Ou et al., 2014; Bojang et al., 2016).

Multiple cross displacement amplification (MCDA) is a novel amplification strategy based on isothermal strand-displacement polymerization reaction (Wang et al., 2015). In MCDA, a set of ten specific primers is designed for each target, with high sensitivity to fg level. In recent years, MCDA has been successfully applied to the detection of various pathogens (Wang et al., 2016a,b). In the previous report, we established an MTB detection method employing MCDA combined with lateral flow biosensor (MCDA-LFB) and preliminarily evaluated its clinical application (Jiao et al., 2019). MCDA-LFB is rapid, sensitive and simple, which is suitable for clinical use and field test. However, the application of LFB needs to open the amplification tube, which might lead to contamination in subsequent experiments. Since late 2019, coronavirus disease 2019 (COVID-19) began to wreak havoc all over the world. Although the COVID-19 pandemic is a setback to TB control, it still brings opportunities. In China, molecular detection platforms, especially techniques based on real-time PCR, are widely used all over the country, which greatly improved the detection ability in basic level hospitals and medical institutions. In this study, we integrated the MCDA assay into real-time PCR platform and assessed its accuracy in clinical pulmonary TB (PTB) cases.

## Materials and Methods

### Study Population

Sputum specimens were prospectively collected from suspected TB patients from January 2019 to March 2019 at the Beijing Chest Hospital. All the enrolled patients had symptoms suggestive of TB and abnormal chest imaging. Each sputum specimen was subjected to smear microscopy, Lowenstein-Jensen (LJ) solid culture, mycobacteria growth indicator tube (MGIT) culture and Xpert (Cepheid, United States) simultaneously. The 1 mL aliquot sputum samples were stored at −80°C for further analysis.

The Ethics Committee approved the study protocol (No. 2020-7). Written informed consent was waived, as the specimens used in this study were leftover sputum samples from the clinical microbiology laboratory.

### Patient Categories

Based on composite reference standard (CRS) which comprises clinical examination, microbiological evaluation, radiological imaging and follow-up data, patients were classified into three groups. (1) Bacteriologically confirmed TB: at least one positive result was obtained from the following tests: smear microscopy, culture or Xpert. (2) Probable TB: no bacteriological evidence of TB was found, but active TB diagnosis was made according to clinical findings, radiological images, treatment response and follow-up data. (3) Non-TB: the alternative diagnosis was established and clinical improvement was achieved without antitubercular treatment.

### Clinical and Laboratory Procedures

Demographic information and clinical data of the enrolled subjects, including age, gender, treatment status, underlying diseases, were collected according to the medical records. The sputum specimens were usually collected early in the morning. Each specimen was transported to the lab within 4 h of collection. A direct smear was prepared and examined by light microscopy after auramine staining. About 2 mL sputum sample was decontaminated with N-acetyl-L-cysteine-sodium hydroxide (NALC-NaOH). After neutralization with sterile saline phosphate buffer (PBS, pH6.8) and centrifugation, the pellet was then inoculated into solid Lowenstein-Jensen medium (Encode Medical Engineering Co., Ltd) and liquid medium using MGIT 960 system (Becton Dickinson, United States). All positive cultures were further confirmed using MPT64 antigen testing (Genesis Biodetection and Biocontrol Co., Ltd).

Xpert (Cepheid) was performed according to the manufacturer’s instructions. In brief, 1–2 mL sputum specimen was mixed with double volumes of Xpert sample processing reagent, and vortexed at 5 min intervals for 15 min. Then 2 mL of the mixture was transferred to the cartridge for Xpert testing.

A glass bead-based kit (CapitalBio Co.) was used to extract the genomic DNA. The defrosted samples were fully digested with 2–4 volumes of 4% NaOH depending on the viscosity of the sputum. After centrifugation, the pellet was washed once using TE buffer and re-suspended with 100 μL DNA extraction buffer. The mixture was transferred to the tube with glass beads and shook for 15 min at a high speed using Extractor 36 (CapitalBio Co.). The tube was heated using a metal rack at 95°C for 5 min. Total 90 μL DNA was obtained after centrifugation at 12, 000 rpm for 5 min and 5 μL of the supernatant was used for MCDA amplification.

The MCDA assay was performed as described previously (Jiao et al., 2019) with some modifications. Two sets of non-labeled primers targeting IS*6110* and IS*1081* were used. To monitor the MCDA reaction, 0.3 μL of EvaGreen dye was added into the reaction mixture to produce the fluorescence signal. The assay was implemented on real-time platform (Agilent AriaMx) using the FAM/SYBR Green channel. The amplification was performed at 67°C for 40 min with plate reading at 30 s intervals. The normalized fluorescence signal no less than 800 and reaction time no more than 35 min were used to determine MCDA positivity. If the normalized fluorescence signal was more than 800 and reaction time more than 35 min, the result was considered to fall in the gray area and the experiment had to be repeated. The result was determined positive if the fluorescence signal remained more than 800 regardless of reaction time and negative if no/less fluorescence signal. The threshold was derived from a preliminary analysis of smear-positive samples and non-TB patients. All laboratory tests were performed with the operator blinded to the clinical information.

### Statistical Analysis

Demographic information of the study population was presented as percentages for categorical variables and medians for continuous variables.

Diagnostic accuracy parameters (sensitivity, specificity, positive and negative predictive values) were calculated using bacteriological results and clinical evidence as reference standards. Among the patients providing more than one sample, diagnostic values were calculated in three ways. (1) Per patient/1st sample, including only the first sample tested for each patient. (2) Per patient/all samples, including all samples tested for each patient and considering the patient as positive if any of these samples was positive. (3) Per sample, including all tested samples individually with performed tests. McNemar’s test was used to compare the differences in sensitivity and specificity. A *P*-value less than 0.05 was considered statistically significant. SPSS version 23.0 software was used for statistical analysis.

## Results

### Study Participants Characteristics

**FIGURE 1:**
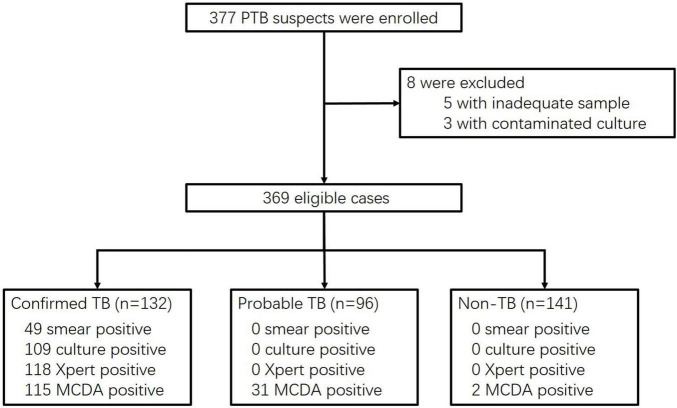
Flow chart of participant recruitment.

Total 377 patients with suspected PTB were enrolled. Eight patients were subsequently excluded due to a lack of adequate samples (*n* = 5) and contaminated cultures (*n* = 3). Thus, 369 patients were included for analysis of MCDA assay diagnostic performance. Figure 1 shows the flow of participants according to the case definition categories.

Overall, 228 patients (61.8%) were diagnosed with active PTB, including 132 with bacteriologically confirmed TB and 96 with probable TB. The 141 non-TB cases (38.2%) were 17 lung cancer patients and 124 patients with other infectious diseases, including four with non-tuberculous mycobacteria (NTM) caused disease. Among NTM patients, two were culture-positive and two were diagnosed by molecular testing of biopsy tissues. The age of patients was younger in the TB group than that in the non-TB group (48.5 years vs. 65 years, *P* < 0.001). TB group had more male patients (75.0% vs. 61.7%, *P* = 0.007). A higher proportion of TB patients had diabetes mellitus compared to non-TB patients (27.2% vs. 6.4%, *P* < 0.001). All patients were HIV-negative. Detailed demographic and clinical data are presented in Table 1.

**TABLE 1:** Demographic and clinical characteristics of the study population.

Characteristic	Total TB (*n* = 228)	Confirmed TB (*n* = 136)	Probable TB (*n* = 92)	Non-TB (*n* = 141)	*P*-value (Total TB vs. Non-TB)
Age, median (range), yr	48.5 (16–88)	48 (17–88)	49 (16–85)	65 (19–97)	<0.001
Gender					
Male, n (%)	171 (75.0)	107 (78.7)	64 (69.6)	87 (61.7)	0.007
Female, n (%)	57 (25.0)	29 (21.3)	28 (30.4)	54 (38.3)	
Treatment status					
New case	174 (76.3)	102 (75.0)	72 (78.3)	/	
Retreated case	54 (23.7)	34 (25.0)	20 (21.7)	/	
Combined extra-pulmonary TB					
Pleural TB	67 (29.4)	32 (23.5)	35 (38.0)	/	
Lymphatic TB	10 (4.4)	5 (3.7)	5 (5.4)	/	
TB meningitis	2 (0.9)	2 (1.5)	0 (0)	/	
Other sites	11 (4.8)	5 (3.7)	6 (6.5)	/	
Underlying diseases					
Diabetes mellitus	62 (27.2)	39 (28.7)	23 (25.0)	9 (6.4)	<0.001
Chronic kidney disease	11 (4.8)	8 (5.9)	3 (3.3)	3 (2.1)	0.188
Autoimmune disease	3 (1.3)	0 (0)	3 (3.3)	2 (1.4)	0.934
Tumor	14 (6.1)	11 (8.1)	3 (3.3)	17 (12.1)	0.046

*Values are No. (%) or as indicated.*

*“/”: not applicable.*

### Performance of Multiple Cross Displacement Amplification Assay in Pulmonary Tuberculosis Diagnosis

In the MCDA detection, six samples corresponding to six patients (three were confirmed TB, the others are probable TB) fell in the gray area. After repeat, four remained the same and two were positive. Thus, all six samples were considered positive in the following analysis.

Diagnostic value was calculated considering the first collected sputum specimen for all enrolled patients (Table 2). Generally, MCDA assay exhibited a sensitivity of 64.0% (146/228) in patients with active PTB, which was significantly higher than Xpert (51.8%, 118/228, *P* = 0.008), culture (MGIT and LJ combined, 47.8%, 109/228, *P* < 0.001) and microscopy (21.5%, 49/228, *P* < 0.001). Then we divided the patients into confirmed TB and probable TB according to CRS without referring to MCDA results. Among 132 confirmed TB cases, the high sensitivity of MCDA assay was shown at 87.1% (115/132), similar to that of Xpert (89.4%, 118/132, *P* = 0.566) and culture (82.6%, 109/132, *P* = 0.303). In addition, the MCDA assay detected 31 probable TB patients. When the MCDA results were integrated into the CRS, these 31 patients were reclassified as confirmed TB cases, and the percentage of confirmed TB increased from 57.9% (132/228) to 71.5% (163/228).

**TABLE 2:** Diagnostic accuracy of different methods in the pulmonary TB patients.

Performance	Microscopy	Culture	Xpert	MCDA	Culture + MCDA
**Sensitivity**					
Confirmed PTB^a^ (*n* = 132)	49/132 (37.1)^b^	109/132 (82.6)	118/132 (89.4)	115/132 (87.1)	127/132 (96.2)^c^
Probable PTB (*n* = 96)	/	/	/	31/96 (32.3)	31/96 (32.3)
PTB total (*n* = 228)	49/228 (21.5)^b^	109/228 (47.8)^b^	118/228 (51.8)^c^	146/228 (64.0)	158/228 (69.3)
Specificity (*n* = 141)	141/141 (100)	141/141 (100)	141/141 (100)	139/141 (98.6)	139/141 (98.6)
PPV total (*n* = 369)	49/49 (100)	109/109 (100)	118/118 (100)	146/148 (98.6)	158/160 (98.8)
NPV total (*n* = 369)	141/320 (44.1)^b^	141/260 (54.2)	141/251 (56.2)	139/221 (62.9)	139/209 (66.5)

*Values are No./total No. (%). MCDA, multiple cross displacement amplification; NPV, negative predictive value; PPV, positive predictive value; Xpert, Xpert MTB/RIF.*

*^a^Patients were classified according to composite reference standard criteria that does not include MCDA results.*

*^b^Statistically significant (P < 0.001) when compared with MCDA assay.*

*^c^Statistically significant (P < 0.05) when compared with MCDA assay.*

**FIGURE 2:**
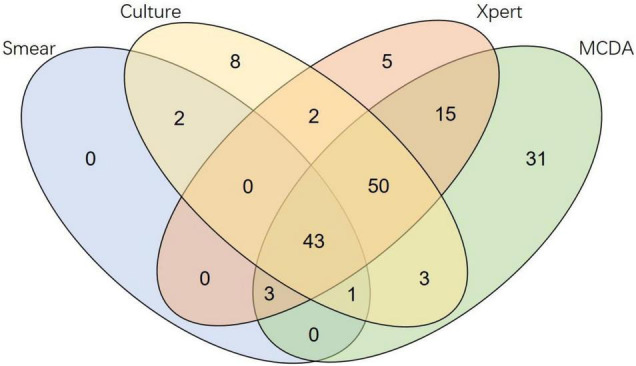
Venn diagram of different diagnostic methods.

A head-to-head comparison of results from all diagnostic methods showed that MCDA assay produced a sensitivity of 89.0% (97/109) in culture-positive patients, 95.9% (47/49) in microscopy-positive cases, and 84.1% in microscopy-negative but culture-positive cases. MCDA assay also identified TB in 35 Xpert-negative cases and 49 culture-negative cases. In microscopy-negative samples, MCDA assay showed a sensitivity of 55.3% (99/179) and Xpert was 40.2% (72/179). MCDA assay was the only positive test in 31 samples, Xpert in 5 samples, and culture in 8 samples (Figure 2).

The specificities of microscopy, culture, Xpert and MCDA assay based on the analysis of the first available sample were 100% (141/141), 100% (141/141), 100% (141/141), and 98.6% (139/141), respectively. With regard to the latter, two lung tumor patients were misdiagnosed as PTB by MCDA assay.

### Per Patient and Per Sample Analysis in Patients With Multiple Samples

A total of 98 patients provided two or more sputum samples, including 188 sputum samples from 86 TB patients and 26 samples from 12 non-TB patients. Per patient sensitivity and specificity analysis used the CRS as the reference standard. Taking only the first available sputum sample into account, the sensitivity of MCDA assay was 60.5% (52/86), while the sensitivity amounted to 75.6% (65/86) when all samples tested by MCDA assay were included in the analysis (Table 3). In comparison, the per patient sensitivity of Xpert, culture and microscopy was 46.5% (40/86), 44.2% (38/86) and 25.6% (22/86) for first available sample, respectively, and 50.0% (43/86), 48.8% (42/86) and 30.2% (26/86) for all samples, respectively. When all tested samples were considered individually, per sample sensitivity showed a sensitivity of 58.5% (110/188) for MCDA assay, which was significantly higher than Xpert (47.9%, *P* = 0.039), culture (44.1%, *P* = 0.005) and microscopy (26.1%, *P* < 0.001). None of the patients in the non-TB group was found to be positive using MCDA assay, Xpert, culture or microscopy. Thus the specificity of each method remained high (100%) either per patient or per sample analysis.

**TABLE 3:** Per patient diagnostic accuracy in the patients with multiple sputum samples.

	Sensitivity	Samples per patient, mean (SD)	Specificity	Samples per patient, mean (SD)
**Microscopy**				
First available sample	22/86 (25.6)^a^	1	12/12 (100)	1
All samples	26/86 (30.2)^a^	2.19 (0.45)	12/12 (100)	2.167 (0.39)
Culture				
First available sample	38/86 (44.2)^b^	1	12/12 (100)	1
All samples	42/86 (48.8)^a^	2.19 (0.45)	12/12 (100)	2.167 (0.39)
Xpert				
First available sample	40/86 (46.5)	1	12/12 (100)	1
All samples	43/86 (50.0)^a^	2.19 (0.45)	12/12 (100)	2.167 (0.39)
MCDA				
First available sample	52/86 (60.5)	1	12/12 (100)	1
All samples	65/86 (75.6)	2.19 (0.45)	12/12 (100)	2.167 (0.39)

*Values are No./total No. (%). MCDA, multiple cross displacement amplification; NPV, negative predictive value; PPV, positive predictive value; Xpert, Xpert MTB/RIF.*

*^a^Statistically significant (P < 0.001) when compared with MCDA assay.*

*^b^Statistically significant (P < 0.05) when compared with MCDA assay.*

## Discussion

Early diagnosis is essential for the control of TB. Although there are many available diagnostic methods for TB, only 59% of PTB cases were bacteriologically confirmed according to WHO report (World Health Organization [WHO], 2021). Alternative test that is rapid, sensitive, specific and easy to perform is always the ideal goal to accelerate the process to end TB. In the previous study, we established a promising method for TB detection using MCDA combined with LFB and its limit of detection was 10 fg (Jiao et al., 2019). With the widespread access to real-time PCR techniques, we further moved to monitor the MCDA products using real-time PCR platform and evaluated its diagnostic accuracy in the PTB cases. As a result, MCDA assay showed a higher overall sensitivity (64.0%), with a difference of more than 10% compared with Xpert (51.8%, *P* < 0.05) and combined culture (MGIT and LJ, 47.8%, *P* < 0.001) for PTB diagnosis. In particular, the sensitivity of MCDA assay was 55.3% (99/179) in smear-negative samples and Xpert was 40.2% (72/179). The difference is in line with a previous publication, where 63% of smear-negative patients were detected by Xpert Ultra and 46% detected by Xpert (Dorman et al., 2018). This indicates that MCDA assay has a similar performance with Xpert Ultra in PTB detection.

In this study, the improved diagnostic value of the MCDA assay was especially pronounced in resolving probable PTB cases. 32.3% (31/96) of cases with negative etiological results (including microscopy, culture or Xpert) were positive for TB using MCDA assay, which notably increased the bacteriological confirmation rate from 57.9% (132/228) to 71.5% (163/228). Therefore, MCDA assay might lead to earlier diagnosis and treatment initiation. In contrast with conventional culture (LJ and MGIT), the MCDA assay detected 49 additional PTB cases (Figure 2). However, it should be noted that 8 samples were detected as positive only by culture, indicating that MCDA assay can be a promising complementary test for PTB diagnosis, but cannot replace the culture-based algorithms.

Examination of multiple specimens from the same TB patient would improve the detection sensitivity of microscopy, culture and Xpert (Bonnet et al., 2007; Wang G. et al., 2018). Wang et al. (Wang G. et al., 2018) reported that the addition of a second Xpert test increased sensitivity by 4.65% among PTB patients, which is similar to this study (3.5%). Our data suggested that the overall sensitivity of the MCDA assay increased with the number of sputum samples tested. Compared with Xpert, culture and microscopy, multiple MCDA assays had a higher incremental yield (15.1%) for detection of PTB patients, with a sensitivity of 60.5% for the first available sample to 75.6% for all samples. Thus, multiple MCDA tests will be beneficial for suspected PTB. In the previous study, a second Xpert assay was not recommended for suspected TB patients, especially in smear-negative cases, considering the high cartridge cost (Wang G. et al., 2018). The cost of MCDA assay is much lower, with only about 1/6 of Xpert per test. In this view, using multiple MCDA tests in suspected TB cases presents an affordable and feasible approach, especially for patients with smear-negative results.

The specificity of the MCDA assay compared to the CRS standard was considered acceptable (98.6%). This was due to two patients being misdiagnosed as PTB by MCDA assay with results confirmed after three repeats. One patient is 76 years old with a malignant tumor of the right lung. The other one is 78 years old with pneumonia caused by Klebsiella pneumonia. We assumed that these two patients could have TB comorbidity or a history of TB, as China is a high TB burden country. A similar situation was reported in other studies, especially when using more sensitive methods, such as Xpert Ultra (Dorman et al., 2018; Wang G. et al., 2020). The Xpert Ultra used multi-copy genes IS*6110* and IS*1081* as the targets and showed increased sensitivity in various kinds of specimen types for TB detection. However, the specificity of Xpert Ultra was lower than Xpert, particularly for patients with a history of TB, as Xpert Ultra might have detected MTB DNA as the remnant of a previous active TB episode (Dorman et al., 2018; Theron et al., 2018). MCDA assay employed the same targets as Xpert Ultra and had high sensitivity, which might also lead to the false-positive result in specificity verification. However, cross-contamination or other unknown reasons cannot be excluded.

Different amplification monitoring methods might influence the sensitivity. Compared with our previous study, MCDA assay using real-time platform did not achieve high sensitivity as that with LFB (64.0% vs. 88.2%) (Jiao et al., 2019). The main reason might lie in the different methods of monitoring the assay products. When the LFB was used to detect the MCDA results, the biotin-labeled MCDA amplicons could form a complex with streptavidin-coated polymer nanoparticles (SA-DNPs) *via* biotin-streptavidin interactions at the conjugated pad, which would amplify the signal and increase the sensitivity. Thus, more paucibacillary patients can be detected using MCDA combined with the LFB platform. Nevertheless, the real-time platform has its advantage. One of the essential steps for reporting MCDA results by LFB is to open the amplification tube, which can produce aerosol droplets containing a high concentration of MCDA products (Wang Y. et al., 2018) and possible contamination presents a significant challenge in this case. In contrast, real-time PCR platform permits monitoring of the fluorescence signal through transparent cover of the unopened tube, thus avoiding possible contamination. This approach is more suitable for areas without specialized product testing laboratories.

Compared to the automated Xpert platform, limitations of the developed MCDA assay should be noted. Firstly, the targets of MCDA assay are multi-copy genes IS*6110* and IS*1081*, which ensure high sensitivity and can even detect IS*6110*-absent strains circulating in Southeast Asia. However, it cannot predict drug resistance of MTB simultaneously. Secondly, MCDA assay needs relatively more manual operations, including extraction of genomic DNA, preparation of reaction mixture, etc. Nevertheless, MCDA assay is suitable for labor-intensive and resource-limited areas as the reagents and consumables are affordable.

In conclusion, this study demonstrated a higher sensitivity of the MCDA assay compared with microscopy, culture or Xpert using sputum samples. MCDA assay has the potential in assisting the accurate and rapid diagnosis of PTB and speeds up initiation of TB treatment in settings equipped with real-time PCR platforms.

## Data Availability Statement

The raw data supporting the conclusions of this article will be made available by the authors, without undue reservation.

## Ethics Statement

The studies involving human participants were reviewed and approved by Beijing Children’s Hospital, Capital Medical University. Written informed consent from the participants’ legal guardian/next of kin was not required to participate in this study in accordance with the national legislation and the institutional requirements.

## Author Contributions

W-WJ conceived and designed the experiments. W-WJ, G-RW, LS, JX, J-QL, Y-CW, and S-TQ performed the experiments. G-RW and H-RH contributed the clinical samples. W-WJ and A-DS contributed the reagents and materials. W-WJ, G-RW, H-RH, and A-DS analyzed the data and wrote the manuscript. All authors have contributed and approved the final version of the manuscript.

## Conflict of Interest

The authors declare that the research was conducted in the absence of any commercial or financial relationships that could be construed as a potential conflict of interest.

## Publisher’s Note

All claims expressed in this article are solely those of the authors and do not necessarily represent those of their affiliated organizations, or those of the publisher, the editors and the reviewers. Any product that may be evaluated in this article, or claim that may be made by its manufacturer, is not guaranteed or endorsed by the publisher.
